# Efficacy and safety of hemoporfin photodynamic therapy in treating port-wine stains in Chinese children: a systematic review and meta-analysis

**DOI:** 10.3389/fped.2024.1501401

**Published:** 2025-01-14

**Authors:** Jing Xu, Hongxin Li

**Affiliations:** Department of Dermatology, Children’s Hospital Affiliated to Capital Institute of Pediatrics, Beijing, China

**Keywords:** port-wine stains, hematoporphyrin monomethyl ether, photodynamic therapy, children, meta-analysis

## Abstract

**Objective:**

The purpose of this study is to explore the efficacy and safety of hematoporphyrin monomethyl ether mediated photodynamic therapy (HMME-PDT) in treating children with port-wine stains (PWS).

**Method:**

Literature related to the topic was searched in PubMed, Embase, Cochrane Library, Web of Science, China National Knowledge Infrastructure, Wanfang, and China Science Technology Journal Database online databases. The quality of the literature was evaluated using the Effective Public Health Practice Project. The I^2^ statistic was used to evaluate the consistency of the results.

**Results:**

A total of 19 papers were included. Meta-analysis showed that more than half of the children (56.3%) achieved efficacy I (improvement ≥ 60%). 17% of children achieved efficacy II (improvement ≥ 75%). Regardless of whether the outcome variable was efficacy I or efficacy II, the therapeutic efficacy in children with PWS aged 0–3 years was superior to those aged 3–6 and 6–18 years, and children who underwent a treatment course of ≥3 sessions showed better outcomes compared to those who have only 1 or 2 sessions. After treatment with HMME-PDT, better efficacy was seen in the PWS of the face and neck and pink/red PWS. Additionally, almost all children with PWS treated with HMME-PDT developed edema (99.9%), more than half presented purpura (67.6%), some developed crust (30.8%) and hyperpigmentation (15.0%), and a few occurred scar (2.4%) and hypopigmentation (1.4%).

**Conclusion:**

After HMME-PDT treatment, more than half of the pediatric patients showed an improvement of ≥60%, and no serious adverse reaction events occurred. This study demonstrated that HMME-PDT possessed promising therapeutic efficacy in children with PWS, suggesting that HMME-PDT could be considered a recommended treatment strategy for pediatric PWS. However, future development of standardized assessment guidelines and comparative studies are needed to validate the aforementioned conclusions.

**Systematic Review Registration:**

https://www.crd.york.ac.uk/prospero/#loginpage, PROSPERO (CRD42024592367).

## Introduction

Port-wine stains (PWS), an alternative name for capillary malformations, is a congenital slow-flow vascular anomaly and one of the most common skin vascular abnormalities (1, 2). PWS usually arises from impaired endothelial cell differentiation and progressive dilation of small venule-like capillaries, with its main characteristic being an increased number of dilated capillaries (3, 4). PWS occurs in 0.1% to 2% of newborns, with no gender predisposition (5). PWS typically manifests in children as flat, light pink, or red macules, which over time may undergo changes such as darkening, thickening, and the development of nodules (6, 7). For instance, the color may intensify and turn purplish in adulthood (8). Throughout the developmental and growth journey, PWS presents a dual harm to affected children. Physiologically, it can result in functional damage, such as hyperplasia of the lips or eyelid thickening that inhibits full closure (9, 10). Additionally, PWS may lead to severe diseases such as glaucoma and delayed visual development, resulting in potential vision loss (6, 11). Psychologically, PWS can instill a sense of stigma in the individuals and inflict distress on the family, profoundly undermining the children's overall well-being and quality of life (12, 13). Therefore, there is an urgent need to identify treatments for children PWS patients to alleviate the disease burden and enhance the overall health of children.

A multitude of treatments for PWS have been employed, such as cryotherapy, surgery, and radioisotope therapy (14). However, these methods often result in noticeable scarring and may even carry a possible risk of skin cancer, leading to their current non-recommendation as the primary treatment option (15). At present, the pulsed dye laser (PDL) is regarded as the gold standard for PWS treatment (16). Yet, with the accumulation of clinical experience, the limitations of PDL have become increasingly evident, characterized by suboptimal therapeutic outcomes and a high likelihood of recurrence (15, 17). An effective and safe treatment approach for PWS remains an unmet need. Photodynamic therapy (PDT) as a promising alternative to PDL has been proposed, particularly with hematoporphyrin monomethyl ether (HMME)-mediated PDT, which has become a primary method for treating PWS in China (15, 18). HMME, also known as hemoporfin, is a new generation of porphyrin photosensitizers (19, 20). Compared to the previous generation of PDT drugs (such as Photofrin® and hematoporphyrin derivative), HMME features a more stable structure, higher photodynamic efficiency, stronger photoactivity, faster clearance rate, and lower toxicity, and it has been widely used in PDT for PWS (21). Nonetheless, there are still inconsistent conclusions regarding the application of HMME-PDT in children. For example, Tan's study suggested HMME-PDT was well-tolerated and effective in Chinese children with PWS, recommending it as the first-line treatment for PWS (22). Whereas Gao et al. concluded that the current evidence was insufficient to support HMME-PDT as a preferred treatment for young children (18).

Based on the above research background, more clinical practice or prospective studies are needed in the future to evaluate and explore the use of HMME-PDT in the children population. To the best of our knowledge, current meta-analyses have mostly focused on comparing different treatment methods for treating PWS in the general population, without simultaneously concentrating on the safety and efficacy of HMME-PDT when applied in children (4, 18, 23). Accordingly, addressing the research gap mentioned, our study aims to comprehensively explore the efficacy and safety of HMME-PDT in treating children with PWS.

## Methods

The research was meticulously planned and executed following the guidelines set forth by the Preferred Reporting Items for Systematic Reviews and Meta-Analyses (PRISMA) framework (24).

### Retrieval strategy

**Figure 1:**
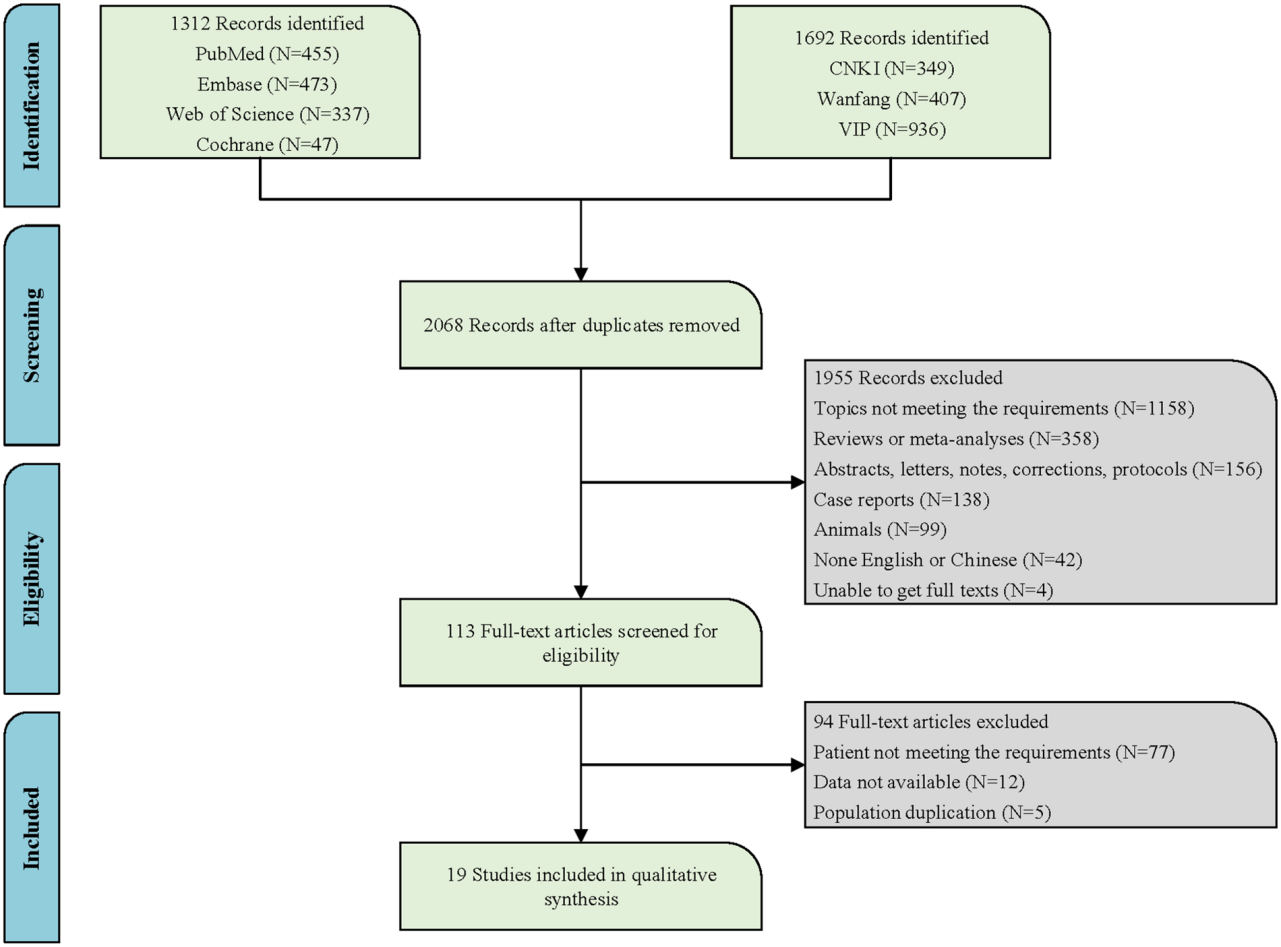
The search flowchart.

Relevant literature up to May 13, 2024, was searched in PubMed, Embase, Cochrane Library, Web of Science, China National Knowledge Infrastructure (CNKI), Wanfang, and China Science and Technology Journal Database (VIP). The primary English search terms were listed below: Port-Wine Stain, Port Wine Stain, Port Wine Stains, Port-Wine Stains, PWS, Nevus Flammeus, Naevus Flammeus, Flame Nevus, Flame Naevus, Nevus Vinosus, Naevus Vinosus, Capillary Malformation, Vascular malformations, Arteriovenous Malformation, Sturge-Weber Syndrome, Sturge Weber Syndrome, Sturge Disease, Sturge Syndrome, Parkes Weber Syndrome, Phakomatosis Pigmentovascularis, hematoporphyrin monomethyl ether, HMME, Hemoporfin, Hematoporphyrins, Hematoporphyrin, Hemedonin, Haematoporphyrin, Photochemotherapy, Photochemotherapies, Photodynamic Therap*, PDT, Photodynamic, Photochemo, Phototherapy, Photosensitizing Agent∗, Photosensitising Agent∗. Detailed search information is provided in Figure 1 and specific English search formulas from the PubMed database are shown in the supplementary material (Supplementary Table S1).

### Study selection and data extraction

The investigation involved the process of initial curation of the literature by importing identified documents into EndNote X9, followed by an initial selection based on the examination of titles and abstracts. Thereafter, a thorough review of the full texts was conducted in accordance with the specified inclusion and exclusion criteria, leading to the exclusion of documents that did not align with the study's standards. The final corpus of literature that met all criteria was subsequently incorporated into the research. Inclusion criteria were determined based on the PICO (Population, Intervention, Comparison, and Outcome) principle to identify articles suitable for this research: (1) Study population: Children (≤18 years) with PWS; (2) Intervention methodology: HMME-PDT; (3) Outcome indicators: Efficacy (or improvement) was the primary outcome, which was quantified as any enhancements to the PWS, such as clearance, fade, and improvement expressed as percentage ranges or alterations of the erythema index (EI); Security evaluation indicators were edema, crust, scar, purpura, hyperpigmentation, and hypopigmentation. Exclusion criteria: (1) The subjects were a mixed age study of children and adults; (2) Data in the literature could not be extracted, e.g., efficacy only qualitatively described, non-60% or 75% cut points; (3) The article was a meta-analysis, review, analysis, case report, conference, letter, errata, program, and was not an English or Chinese article; (4) Where there was overlap in the study population, only the latest or most complete literature was included.

In this survey, information such as the authors of the literature, publication years, countries of publication, types of studies, sample sizes, ages, genders, pretreatment methods, the location and size of the PWS, as well as detailed treatment approaches were collected based on the research requirements. Additional data can be found in Supplementary Table S2.

### Outcome measure

To facilitate the meta-analysis, we categorized the improvement effects reported in the literature into two main groups: Efficacy I and Efficacy II. Efficacy I referred to a ≥60% improvement in PWS from before to after treatment. Efficacy II indicated a ≥75% improvement in PWS from before to after treatment. The meta-analysis included percentage ranges that could potentially be translated into categorical scales, as well as other percentage ranges.

The primary outcome measure was assessed by taking photographs of the children before and after each treatment session, based on the regression of the lesions. The methods employed included conventional photography as well as Vein Illumination Skin Analysis (VISIA) skin imaging system. Conventional photography involved taking pictures of the patient before and after each treatment. Three researchers independently evaluated the therapeutic effect based on the pre-and post-treatment photographs, assessing the treatment outcome by the degree of lesion resolution (25). VISIA involved taking standard digital photographs before and after treatment under identical camera settings and lighting conditions from various angles. The images were then analyzed using Image J software to measure the EI within the images, with the treatment efficacy evaluated by the percentage decrease in EI values (25).

### Literature quality assessment methodology

The Effective Public Health Practice Project (EPHPP) tool was employed to evaluate the quality of the literature, assessing selection bias, study design, confounders, blinding, data collection methods, and withdrawals and dropouts—six key areas in total (26). Each area was graded as strong, moderate, or weak, with the overall study rating determined by these assessments. Following the EPHPP guidelines, studies without any weak grades and with four or more strong grades were categorized as “strong”; those with fewer than four strong grades and one weak grade were deemed “moderate”; and those with two or more weak grades were labeled “weak”.

### Statistical analysis

The “RATE” was utilized as the effect measure, with the effect size expressed as a 95% confidence interval (CI). Notably, in this article, RATE denoted the pooled estimate of the proportion of patients who achieved the efficacy I (or II) and those who experienced the aforementioned adverse side effects. The 95% CI indicated that if multiple samples were repeatedly drawn from the same population and a confidence interval was calculated for each sample, then approximately 95% of these intervals would contain the true value of the population parameter. Due to the high heterogeneity observed in the majority of outcomes, a random-effects model was employed for all analyses. Improvements were also analyzed stratified by age (0–3/3–6/6–18 years old), sex (male/female), session (1/2/≥3), location (face/neck/trunk or extremities), and type (pink/red/purple or hypertrophic). All studies were statistically analyzed using Stata 15.1 software (StataCorp, College Station, TX, USA), with a difference considered statistically significant at *P* < 0.05.

### Inclusion of literature and study characteristics

Following the search strategy in both Chinese and English databases, a total of 3,004 articles were retrieved. After removing duplicates, 2,068 articles remained. Based on the review of titles and abstracts, 1,158 articles were excluded due to topics not meeting requirements. A further 358 articles were excluded as they were reviews or meta-analyses. Additionally, 156 records that were merely abstracts, letters, notes, corrections, or protocols were also eliminated. One hundred and thirty-eight case reports, 99 articles of animal experiments, 42 non-English articles, and 4 articles for which the full text could not be obtained were all excluded. After the aforementioned screening process, 113 articles remained. Subsequently, upon reviewing the full texts of the remaining articles, 94 were excluded due to ineligible patient criteria, data not available, and duplicate populations, leaving a final inclusion of 19 articles (17, 20, 22, 25, 27–41). Figure 1 illustrates the specific search flowchart. All studies were conducted in the Chinese population, involving a total of 5,859 children with PWS. Four studies were prospective, and 15 were retrospective. All studies provided specific details regarding the treatment, with 73.68% of the studies (14/19) detailing the exact treatment locations, and 52.63% of the studies (10/19) providing information on the types of PWS. Detailed information is presented in Supplementary Table S2.

### Evaluation of bias risk

As shown in Table 1, the quality of the included studies was assessed using the EPHPP tool. According to the tool's guidelines (with strong, moderate, and weak ratings for each item), one study with no weak ratings and four strong ratings was classified as strong. Fifteen studies with fewer than four strong ratings and one weak rating were rated as moderate, and three studies with two or more weak ratings were classified as weak. Overall, the methodological quality is within an acceptable range.

**Table 1:** Quality assessment of involved studies.

Study	Selection bias	Study design	Confounders	Blinding	Data collection methods	Withdrawals and drop-outs	Global rating
Strong	Representative of target population and 80% participation	RCT or CCT	Controlled for at least 80% of confounders	Assessor and participants blinded	Valid and reliable tools	80% or more at follow-up	No WEAK ratings
Moderate	Likely to be representative and 60–79% participation	Cohort analytic, case control or interrupted time series	Controlled for 60- 70% of confounders	Assessor or participants blinded	Valid tools but reliability not measured or described	60%–79% at follow up	One WEAK rating
Weak	Not likely to be representative and <60% participation	Other designs	Controlled for <60% of confounders	Assessor and participants aware	Validity and reliability not measured or described	<60% at follow up	Two or more WEAR ratings
Yu 2024	Moderate	Moderate	Strong	Weak	Strong	Strong	Moderate
Chai 2023	Weak	Moderate	Strong	Moderate	Moderate	Strong	Moderate
Chen 2023	Moderate	Moderate	Strong	Moderate	Moderate	Strong	Moderate
Huang 2023	Moderate	Moderate	Strong	Moderate	Moderate	Strong	Moderate
Sun 2023	Strong	Moderate	Strong	Moderate	Strong	Strong	Strong
Wang 2023	Strong	Moderate	Weak	Weak	Moderate	Strong	Weak
Zhang 2023_1	Strong	Moderate	Moderate	Moderate	Strong	Strong	Moderate
Zhang 2023_2	Moderate	Moderate	Strong	Moderate	Moderate	Strong	Moderate
Zhu 2023	Moderate	Moderate	Strong	Moderate	Moderate	Strong	Moderate
Liu 2022	Strong	Moderate	Strong	Moderate	Moderate	Strong	Moderate
Peng 2022	Weak	Moderate	Strong	Moderate	Moderate	Strong	Moderate
Tao 2022	Strong	Moderate	Weak	Weak	Moderate	Strong	Weak
Zhang 2022_1	Moderate	Moderate	Strong	Moderate	Moderate	Strong	Moderate
Zhang 2022_2	Strong	Moderate	Strong	Moderate	Moderate	Strong	Moderate
Huang 2021	Moderate	Moderate	Strong	Moderate	Moderate	Strong	Moderate
Tan 2021	Strong	Moderate	Strong	Moderate	Moderate	Strong	Moderate
Khalaf 2020	Weak	Moderate	Strong	Moderate	Moderate	Strong	Moderate
Zhang 2019	Strong	Moderate	Weak	Weak	Moderate	Strong	Weak
Zhang 2014	Strong	Moderate	Moderate	Moderate	Moderate	Strong	Moderate

RCT, randomized controlled trial; CCT, controlled clinical trial.

### Efficacy and stratification analysis of HMME-PDT in the treatment of children with PWS

**Figure 2:**
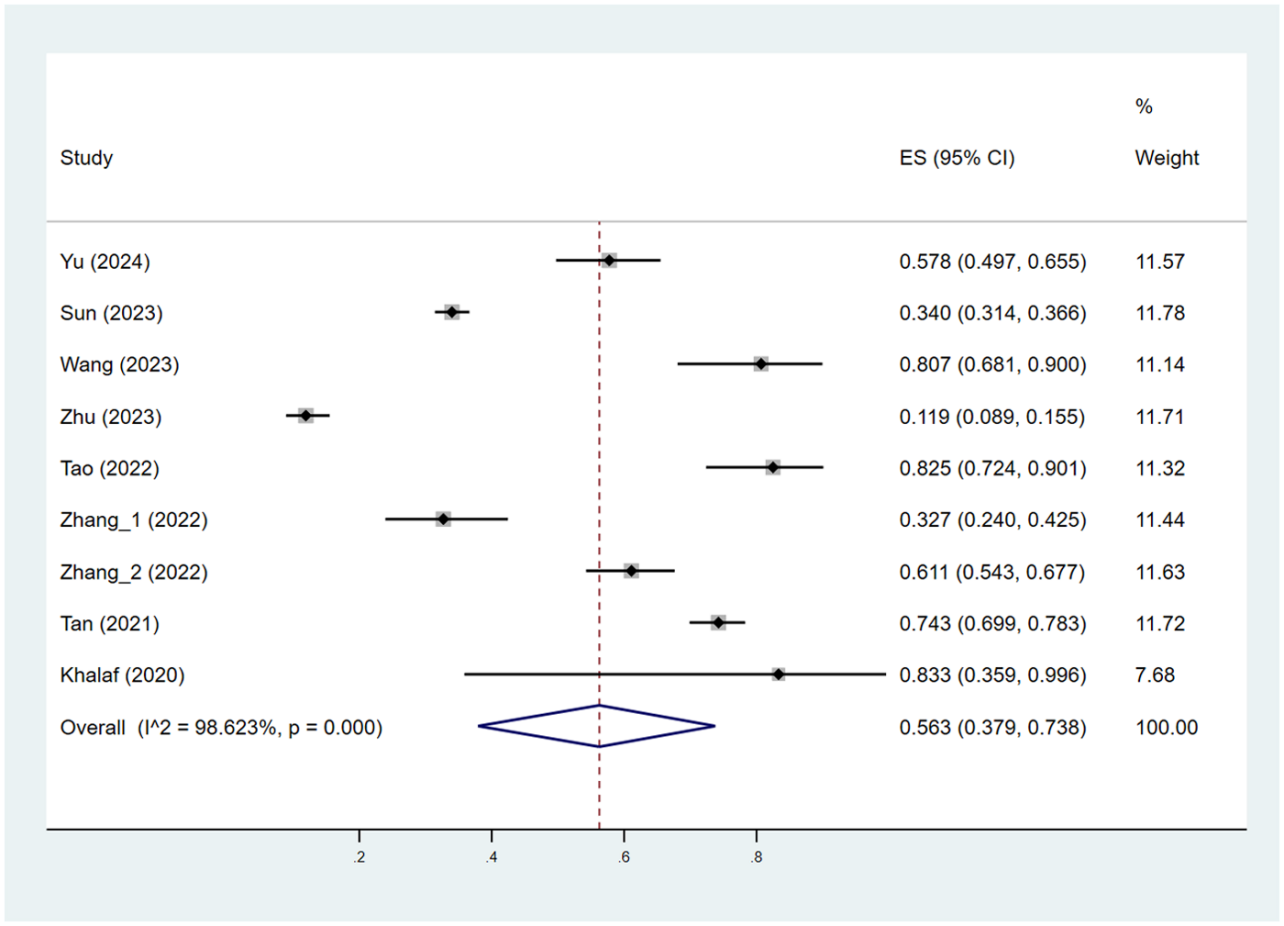
Pooled estimates of the proportion of patients achieving efficacy I (improvement ≥ 60%). ES indicated RATE; CI indicated 95% confidence interval.

**Figure 3:**
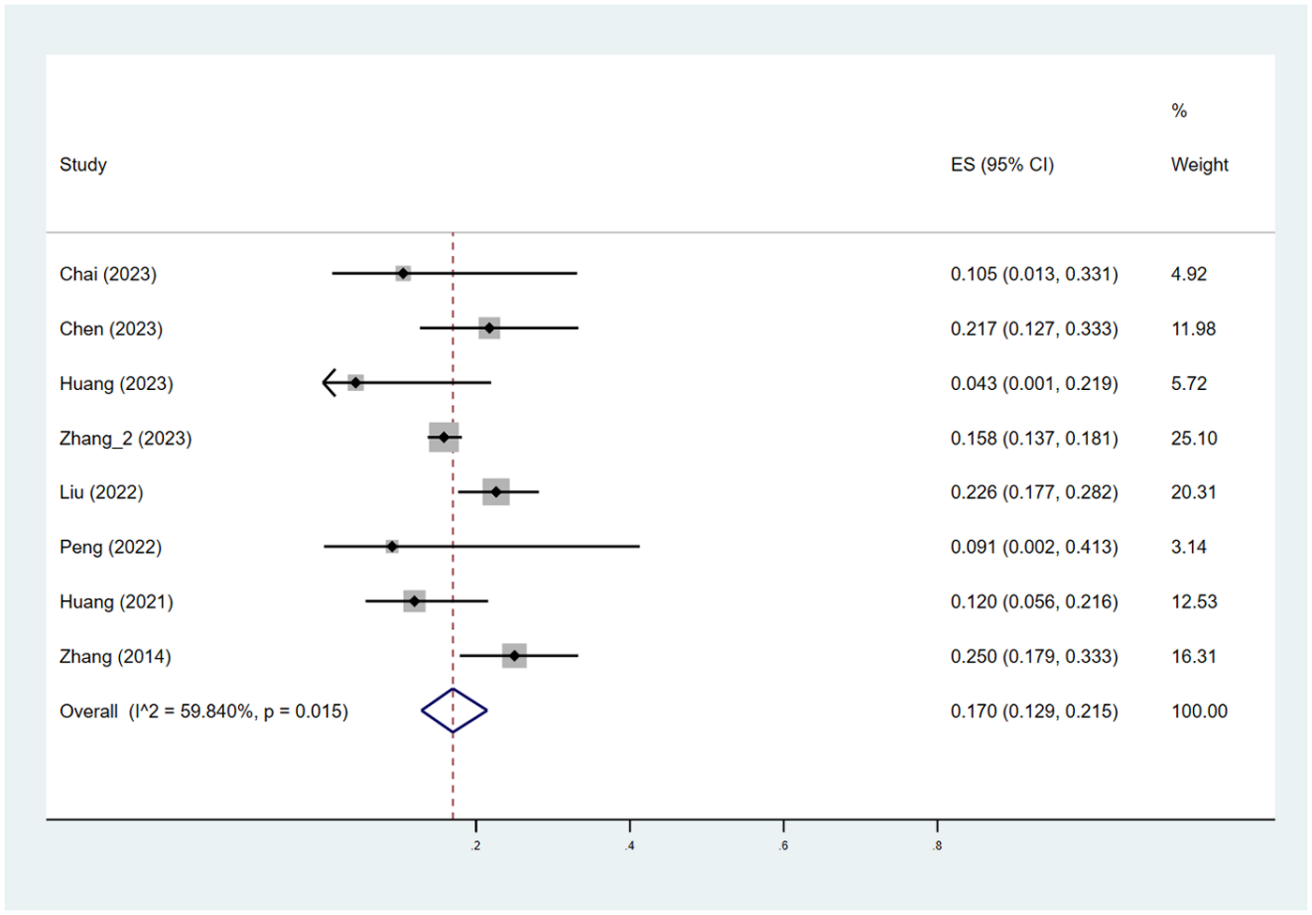
Pooled estimates of the proportion of patients achieving efficacy II (improvement ≥ 75%). ES indicated RATE; CI indicated 95% confidence interval.

As depicted in Figures 2, 3, the pooled estimate of the proportion of patients achieving efficacy I (improvement ≥ 60%) was 56.3%. The pooled estimate of the proportion of patients achieving efficacy II (improvement ≥ 75%) was 17.0%.

A meta-regression analysis was conducted to explore the sources of heterogeneity among groups with efficacy I and efficacy II, stratified by age, gender, treatment course, location, and type. As shown in Supplementary Table S3, among patients achieving efficacy I, the “location” may be a source of study heterogeneity (*P* < 0.05), while all others were not associated with between-study heterogeneity (*P* > 0.05).

As shown in Table 2, the pooled estimate proportions of patients achieving efficacy I across the age groups were 56.1% for those aged 0–3 years, 51.3% for those aged 3–6 years, and 47.0% for those aged 6–18 years. The pooled estimated proportions of patients achieving efficacy II were 18.5% for the 0–3 years age group, 15.1% for the 3–6 years age group, and 15.2% for the 6–18 years age group.

**Table 2:** Efficacy and stratification analysis of HMME-PDT for the treatment of PWS in children.

Outcome indicators	Included studies	n/N	RATE (95% CI)	*I* ^2^
Improvement ≥ 60%				
Overall	9	1,196/2,777	0.563 (0.379, 0.738)	98.62
Age (years)				
0–3	6	579/1,369	0.561 (0.341, 0.769)	97.87
3–6	4	163/326	0.513 (0.130, 0.887)	98.15
6–18	5	94/245	0.470 (0.179, 0.771)	94.81
Sex				
Male	2	185/276	0.672 (0.616, 0.727)	NA
Female	2	273/382	0.716 (0.669, 0.760)	NA
Session				
1	2	128/254	0.503 (0.442, 0.565)	NA
2	2	101/148	0.692 (0.615, 0.765)	NA
≥3	3	615/1,524	0.641 (0.242, 0.957)	NA
Location				
Face	4	480/656	0.704 (0.568, 0.824)	91.17
Neck	2	54/59	0.923 (0.834, 0.984)	NA
Trunk/extremities	2	22/84	0.259 (0.169, 0.361)	NA
Type				
Pink	3	91/123	0.737 (0.620, 0.840)	NA
Red	2	298/385	0.781 (0.737, 0.821)	NA
Purple/hypertrophic	3	102/212	0.443 (0.321, 0.568)	NA
Improvement ≥ 75%				
Overall	8	291/1,670	0.170 (0.129, 0.215)	59.84
Age (years)				
0–3	3	79/393	0.185 (0.132, 0.245)	NA
3–6	2	86/561	0.151 (0.122, 0.182)	NA
6–18	5	76/486	0.152 (0.075, 0.246)	68.01
Sex				
Male	2	30/129	0.224 (0.153, 0.303)	NA
Female	2	30/155	0.174 (0.113, 0.243)	NA
Session				
1	4	50/343	0.069 (0.000, 0.226)	92.82
2	5	31/250	0.092 (0.029, 0.178)	69.00
≥3	2	40/127	0.310 (0.232, 0.394)	NA
Localization				
Face	4	91/385	0.190 (0.112, 0.281)	66.26
Neck	1	4/19	0.211 (0.061, 0.456)	NA
Trunk/extremities	1	0/31	0.000 (0.000, 0.112)	NA
Type				
Pink	1	11/26	0.423 (0.234, 0.631)	NA
Red	3	64/302	0.196 (0.128, 0.273)	NA
Purple/hypertrophic	3	18/88	0.170 (0.062, 0.309)	NA

n/N, Number of positive cases/sample size; CI, Confidence interval. Rate represented the pooled estimate of the proportion of patients who achieved efficacy I (improvement ≥ 60%) or efficacy II (improvement ≥ 75%); *I*^2^ represented the statistic of heterogeneity test statistics between each study.

Subsequently, a stratified analysis was conducted based on gender. The study found that in the male patients, the pooled estimated proportions of patients achieving efficacy I or efficacy II were 67.2% and 22.4%, respectively. Similarly, in the female, the pooled estimated proportions of patients achieving efficacy I or efficacy II were 71.6% and 17.4%, respectively.

As shown in Table 2, the analysis was conducted by categorizing the treatment courses into three groups: one course, two courses, and three or more courses. The pooled estimated proportions of patients achieving efficacy I or efficacy II after one course of HMME-PDT treatment were 50.3% and 6.9%, respectively. After two courses of treatment, the pooled estimated proportions of patients achieving efficacy I or efficacy II were 69.2% and 9.2%, respectively. After three or more courses of treatment, the pooled estimated proportions of patients achieving efficacy I or efficacy II were 64.1% and 31.0%, respectively.

The data were segmented and analyzed according to the regions of the face, neck, and trunk/extremities. Patients with PWS located on the face, neck, and trunk (or limbs) who underwent HMME-PDT treatment had pooled estimated proportions achieving efficacy I as follows: 70.4%, 92.3%, and 25.9%, respectively. Additionally, the pooled estimated proportions of patients with PWS located on the face, neck, and trunk (or limbs) who achieved efficacy II after HMME-PDT treatment were 19.0%, 21.1%, and 0.0%, respectively.

Finally, a stratified analysis was performed based on the types of PWS, categorizing the study subjects into three groups: pink, red, and purple/hypertrophic PWS. Patients with pink, red, and purple (or hypertrophic) PWS who achieved efficacy I after treatment had pooled estimated proportions of 73.7%, 78.1%, and 44.3%, respectively. Furthermore, the pooled estimate of the proportions of patients with the aforementioned types of PWS who achieved efficacy II after treatment were 42.3%, 19.6%, and 17.0%, respectively.

### Adverse reactions

As seen in Table 3, nearly all children (99.9%) presented with edema, over half of the children (67.6%) experienced purpura, 30.8% of children developed crusting and 15.0% of children developed hyperpigmentation. Additionally, a very small number of children exhibited scarring (2.4%) and hypopigmentation (1.4%). Overall, no severe adverse reactions were reported.

**Table 3:** Adverse effects of HMME-PDT for the treatment of PWS in children.

Indicators	Included studies	n/N	RATE (95% CI)	*I* ^2^
Edema	7	1,094/1,103	0.999 (0.991, 1.000)	48.08
Crust	7	263/1,103	0.308 (0.144, 0.501)	97.40
Scar	7	27/1,108	0.024 (0.009, 0.045)	65.23
Purpura	3	207/573	0.676 (0.128, 1.000)	99.15
Hyperpigmentation	6	343/985	0.150 (0.003, 0.434)	98.90
Hypopigmentation	4	7/446	0.014 (0.004, 0.029)	0.00

n/N, Number of positive cases/sample size; CI, Confidence interval. Rate represented the pooled estimate of the proportion of patients who occurred adverse effect; *I*^2^ represented the statistic of heterogeneity test statistics between each study.

## Discussion

PWS not only inflicts physical and psychological harm on children but also severely impacts the quality of life of their families (42, 43). Although PDL is considered a first-line treatment for PWS, there is currently no unified international standard for the treatment of children with PWS (44). HMME-PDT, a novel treatment method for PWS recently proposed in China, holds great potential (45). Consequently, this study explored the efficacy and safety of HMME-PDT in the children PWS population, with the main conclusions as follows: (1) More than half of the children had efficacy I (improvement ≥ 60%), but less than 20% of children achieved efficacy II (improvement ≥ 75%). (2) The younger the age at which treatment was administered, the better the therapeutic outcome. Furthermore, a greater number of treatment courses, specifically more than three, may yield improved efficacy. Additionally, treatment of PWS located on the face and neck, as well as those with lighter coloration, may demonstrate better outcomes. (3) After HMME-PDT treatment, virtually all children with PWS exhibited edema, over half developed purpura, some experienced crust and hyperpigmentation, and a minority presented with scar and hypopigmentation.

Over half of the children showed an improvement of more than 60%, demonstrating that HMME-PDT possessed a certain efficacy in the treatment of children with PWS. This was consistent with the findings of Chai and Tan, both of whom concluded that HMME-PDT showed good potential in the treatment of children with PWS (22, 27). Age was closely related to the growth and development of PWS (28). The study found that in both efficacies I and II groups, the proportion of efficacy in children aged 0–3 years was higher than in other age groups. The above result was similar to that of Lin et al., who found that efficacy decreases with age (46). Age has been correlated with the color, thickness, and nodularity of PWS lesions, with children tending to have thinner skin with better irradiation penetration and therefore better treatment outcomes (4, 47). Moreover, when analyzing the data by gender, observing the proportion of females achieving efficacy I (improvement ≥ 60%) was higher than that of males. However, in the efficacy II group (improvement ≥ 75%), a higher proportion of males was noted. Tan's study found that females had better treatment outcomes (22). Yet, in reality, few scholars have focused on the gender differences in HMME-PDT treatment for children with PWS, and conclusions regarding gender differences in efficacy cannot be firmly established. Therefore, future clinical or prospective studies are needed to examine the differences in treatment outcomes related to gender.

Consistent with the research by Huang and Chen et al., our study found that children with PWS who underwent a greater number of HMME-PDT treatment courses experienced better therapeutic outcomes (28, 30). The study by Lin et al. also indicated that the efficacy of 2 consecutive treatments was significantly higher than that of a single one (46). Furthermore, a subgroup analysis was conducted on the efficacy of PWS treatment at different anatomical locations. The results indicated that PWS located on the face and neck responded better than extremities or trunk, with a higher proportion of children showing improved outcomes. It was hypothesized that this may be due to the generally thinner skin on the face and neck, allowing light to penetrate more easily and thus facilitating the resolution of the lesions following treatment (4, 48). Similar to the research by Sun et al., their study found that the therapeutic effect of HMME-PDT was greatly influenced by the location of the PWS (35). But somewhat differently from ours, they believed that the efficacy on the face was not very effective. Furthermore, Zhang's research suggested that there was no significant difference in the therapeutic effect of PWS on the face, neck, and other parts of the body (17). The aforementioned differences were likely attributable to variations in the study populations, and the sample size also exerted a certain degree of influence. Ultimately, stratified analysis based on different types of PWS revealed that, consistent with the research by Zhang et al., purple or hypertrophic PWS types respond less favorably to HMME-PDT compared to pink or red types (17). It is widely acknowledged that the depth and thickness of blood vessels are the most critical factors affecting the treatment outcomes of PWS, with purple PWS often presenting thicker nodules, thus leading to poorer therapeutic results (15, 30).

In this survey, although almost all children experienced edema following HMME-PDT treatment, no severe adverse events were observed. Moreover, minor adverse reactions were likely to occur in most studies. For instance, in the research by Li et al., the side effects following HMME-PDT treatment were primarily manifested as edema, crusting, and excessive pigmentation (49). Besides, edema has been reported to be common in children and adults after PDT (4). In addition, the occurrence of purpura after treatment is also a normal and anticipated reaction, as it typically signifies that the abnormal blood vessels have been damaged and do not require special treatment (50). However, if further reactions occur, such as the skin turning grayish, medical intervention may be necessary (51). Hyperpigmentation and hypopigmentation also occurred partially in the children's population in this study, but there was no cause for concern as these reactions usually subsided within 2–6 months (48). Alternatively, concerning the recurrence of PWS, statistical analysis could not be performed due to the absence of data on recurrence in the original literature. In the included literature, studies by Tan (eight weeks after treatment), Zhang (with an average follow-up of 21.3 months), and Yu (with follow-ups ranging from 2–3 years) demonstrated no observed recurrences during the follow-up periods after HMME-PDT treatment (22, 25, 39). This suggested to some extent that the capillaries in PWS may suffer permanent damage that was difficult to repair after HMME-PDT, thereby preventing the recurrence of the condition (22). Although there was a study reporting no recurrences over an 18-year follow-up period post-PDT treatment, long-term follow-up studies specific to HMME-PDT were relatively scarce (52). Future long-term studies are essential to confirm the recurrence and progression of PWS in children patients treated with HMME-PDT. In summary, the aforementioned conclusions indicated that adverse events associated with HMME-PDT treatment for children with PWS were mild and transient. HMME-PDT is relatively safe for children with PWS. However, in the future, clinicians should consider taking appropriate measures early on when applying HMME-PDT to treat children with PWS, in order to address the mentioned reactions and safeguard the satisfaction of the patients as much as possible.

HMME, as a second-generation photosensitizer, possesses enhanced photodynamic effects, higher targeting specificity, lower toxicity, and reduced skin phototoxicity (25). In our study, HMME used as a photosensitizer in PDT for pediatric patients with PWS, demonstrated relatively good improvement effects. Most studies also suggested that HMME-PDT was a safe and effective method for the clinical treatment of PWS, indicating that HMME had certain advantages in treating PWS-related lesions to a certain extent (25, 41). There exist photosensitizers with longer absorption wavelengths, such as indocyanine green (ICG) (53). Although studies have shown that ICG may be a promising photosensitizer for PDT, its application in the treatment of PWS is currently very limited in clinical practice and remains in the stage of clinical research and exploration (54). Significantly, while longer wavelengths are more effective in targeting deeper lesions, the current focus on the development and application of photosensitizers is predominantly in oncological treatments, with a scarcity of exploration in the context of PWS (55). Moreover, current research on various photosensitizers and their penetration depth in the treatment of PWS was very limited. Future scholars should focus on this area to promote further advancement in the field of PWS treatment.

Additionally, all cases of HMME-PDT for PWS treatment have been conducted in China, and our study has shown promising efficacy in children (18). Numerous studies have also demonstrated satisfactory outcomes across various populations. For instance, a study by Sun et al., which included 2,952 subjects aged from 8 months to 56 years, indicated that HMME-PDT for PWS is highly effective, with high cure rates and mild local reactions, making it a preferred treatment method (35). Yu et al.'s research similarly confirmed the effectiveness, safety, and good patient tolerance of HMME-PDT for PWS (25). However, Gao et al.'s study suggested that, across all age groups, the clearance rate of PDL was generally higher than that of HMME-PDT (18). PDL exerts its effect by emitting yellow light (such as 585 nm or 595 nm), which is preferentially absorbed by hemoglobin, leading to the selective closure of dilated capillaries in the upper dermis and subsequently causing the color of PWS to gradually lighten (56). Studies have shown that during PDL treatment, while blood vessels are damaged, local hypoxia is also induced, leading to the upregulation of hypoxia-inducible factor (HIF-1α) (19). HIF-1α triggers the transcription of vascular endothelial growth factor (VEGF) (57). VEGF, through the VEGF receptor-2 (VEGFR-2) signaling pathway, regulates angiogenesis, ultimately leading to the revascularization and reconstitution of blood flow in PWS vessels (58, 59). However, HMME-PDT involves the intravenous administration of the photosensitizer HMME, which accumulates and resides within the dilated capillaries (34). Subsequently, light of an appropriate wavelength is applied to generate oxygen-derived free radicals, selectively destroying the vascular walls of PWS without harming the surrounding tissues, thereby exerting a more effective therapeutic action (60). Therefore, although PDL is currently the standard treatment for PWS, recent studies have pointed out recurrences and patient resistance, leading to suboptimal treatment progress (61). Some studies even recommend HMME-PDT as the first-choice treatment for PWS over PDL (22). Peng et al.'s single-center retrospective study also indicated that HMME-PDT may be more effective in treating purple and red PWS than PDL, with the overall treatment effect for purple PWS being greater than that for red PWS (34). Notably, HMME-PDT requires high technical proficiency from physicians, and precise operation is necessary to avoid suboptimal outcomes. Therefore, HMME-PDT should be applied under the guidance of experienced doctors, adhering to expert consensus (62). In summary, given the ongoing debate about the efficacy and safety of HMME-PDT, larger-scale studies are needed in the future to further verify its exact effects in treating PWS.

This study addresses the efficacy as well as the safety of HMME-PDT for the treatment of PWS in children, for which, according to the search, there are no targeted studies of the mentioned research field for the time being. We expect this review to provide strong evidence for the medical care of children with PWS and to help reduce the physical and psychological damage to children as well as the emotional burden on families. However, our study also has certain limitations. Firstly, previous treatment history and the size of the lesions could potentially affect the therapeutic outcomes, but due to the limitations of the original literature, it was not possible to conduct a detailed analysis. Secondly, the majority of the included studies reported short-term efficacy, with a lack of long-term outcomes. Thirdly, all HMME-PDT studies conducted thus far have been carried out in China, hence caution should be exercised when generalizing these findings to other populations. Hence, more high-quality clinical studies dedicated to exploring the value of HMME-PDT for the treatment of children with PWS are needed in the future.

## Conclusion

The meta-analysis discovered that over half of the children treated with HMME-PDT for PWS had an improvement ≥ 60% (efficacy I), with better outcomes observed in younger children who received longer courses of treatment, and facial/neck PWS and pink/red PWS improved better after treatment. Although most patients experienced edema after HMME-PDT therapy, there were no severe adverse reactions. Future large-scale, comprehensive prospective studies could be employed to validate the aforementioned findings, and clinicians should consider a multitude of factors when applying HMME-PDT for the treatment of children with PWS.

## Data Availability

The raw data supporting the conclusions of this article will be made available by the authors, without undue reservation.
