# Supplementary material for: Efficacy and safety of hemoporfin photodynamic therapy in treating port-wine stains in Chinese children: a systematic review and meta-analysis
Source: Front Pediatr. 2025 Jan 14;12:1501401. doi: 10.3389/fped.2024.1501401 (PMC11772295; doi:10.3389/fped.2024.1501401)
Supplement: Supplementary file 1 [file Table1.docx]

**Table S1 The search strategy of meta-analysis**

| **Items** | **Contents** |
| --- | --- |
| PubMed search form | ((((((((((((((((((((Port-Wine Stain) OR (Port Wine Stain)) OR (Port Wine Stains)) OR (Port-Wine Stains)) OR (PWS)) OR (Nevus Flammeus)) OR (Naevus Flammeus)) OR (Flame Nevus)) OR (Flame Naevus)) OR (Nevus Vinosus)) OR (Naevus Vinosus)) OR (Capillary Malformation)) OR (Vascular malformations)) OR (Arteriovenous Malformation)) OR (Sturge-Weber Syndrome)) OR (Sturge Weber Syndrome)) OR (Sturge Disease)) OR (Sturge Syndrome)) OR (Parkes Weber Syndrome)) OR (Phakomatosis Pigmentovascularis)) AND ((((((((((((((((hematoporphyrin monomethyl ether) OR (HMME)) OR (Hemoporfin)) OR (Hematoporphyrins)) OR (Hematoporphyrin)) OR (Hemedonin)) OR (Haematoporphyrin)) OR (Photochemotherapy)) OR (Photochemotherapies)) OR (Photodynamic Therap*)) OR (PDT)) OR (Photodynamic)) OR (Photochemo)) OR (Phototherapy)) OR (Photosensitizing Agent∗)) OR (Photosensitising Agent∗)) |

**Table S2 Basic information on the inclusion of studies**

| Author | Year | Country | Setting | Study design | Sample size | Sex (M/F) | Age, y | Pre-treatment | Location | Type | Size/ cm^2^ | Therapy | Treatment sessions | Treatment intervals, m | Fitzpatrick skin type | Cooling | FU, m | Outcomes |
| --- | --- | --- | --- | --- | --- | --- | --- | --- | --- | --- | --- | --- | --- | --- | --- | --- | --- | --- |
| Yu | 2024 | China | 2017.03-2020.06 | Retrospective | 161 | - | 1.5 - 17.9 | - | - | - | - | 5 mg/kg injected intravenously within 20 min, 532-nm green LED light 10 min after infusion for 20-25 min, power density ranging 80-85 mw/cm^2^, energy density of 96-115 J/cm^2^ | - | - | - | Cold air when necessary | - | Improvement, edema, crust, hyperpigmentation, hypopigmentation |
| Chai | 2023 | China | 2021.01-2023.01 | Prospective | 19 | - | < 6 8, 6-15 11 | - | Face | - | - | 5 mg/kg injected intravenously for 20 min, 532-nm green LED light 10 min after infusion for 20-22 min, power density ranging 80-110 mw/cm^2^, energy density of 105-125 J/cm^2^ | - | - | - | Intermittent ice compress for at least 30 min | 2 | Improvement |
| Chen | 2023 | China | 2017.07-2022.07 | Retrospective | 69 | - | 2-5 30, 6-18 39 | - | - | - | - | 5 mg/kg injected intravenously within 20 min, 530±10 nm LED light 10 min after infusion for 20 min, power density ranging 80-110 mw/cm^2^ | ≥ 2 | - | - | A local cooling spray device when obvious pain | - | Improvement |
| Huang | 2023 | China | 2020.01-2021.12 | Retrospective | 23 | 14/9 | 3.70 ± 6.17 | 14 | Face | Red 21, purple 1, hypertrophic 1 | > 40 | 5-5.5 mg/kg intravenously pumped for 20 min, 532 nm light 10 min after injection for 18-22 min, power density ranging 80-90 mw/cm^2^ | ≥ 2 | 3.81 ± 1.12 | III 19, IV 4 | - | 2 | Improvement, edema, crust, pustules, hyperpigmentation, hypopigmentation |
| Sun | 2023 | China | 2017.03-2022.12 | Retrospective | 2522 | 1234/1288 | < 3 1591, 3-14 931 | PDL 537, PDT 175, PDL+PDT 229, other 113 | Face 2221, neck 377, upper limbs 141, chest 123, head 89, lower limbs 69, back 37, others 4 | Pink 519, purplish red 2003 | - | 5 mg/kg intravenously, 532 nm LED light for 15-20 min, power density ranging 80-100 mw/cm^2^ | - | - | - | Concurrent air coolers during radiation | - | Improvement |
| Wang | 2023 | China | 2017.03-2019.03 | Retrospective | 57 | - | < 3 19, 3-10 34, 11-14 4 | 0 | - | - | - | 5 mg/kg injected within 10 min, 532 nm LED light 5 min after injection for 20 min, power density ranging 80-95 mw/cm^2^, energy density of 96-115 J/cm^2^ | - | 2-3 | - | - | ≥ 6 | Improvement |
| Zhang_1 | 2023 | China | 2019.09-2020.09 | Prospective | 34 | 13/21 | 3.7 ± 2.5 | - | Face | Red | - | 5 mg/kg injected intravenously within 5 min or 20 min, 532 nm green LED light for 20 min, power density of 80 mw/cm^2^, energy density of 96 J/cm^2^ | 3 | 2 | - | Adjustable air fans in the LED machine to keep the temperature under 40°C | 6 | Edema, crust, scar, purpura |
| Zhang_2 | 2023 | China | 2017.03-2019.12 | Retrospective | 1080 | - | < 3 303, 3-6 414, 6-14 363 | PDL 353, other laser 82, other PDT 80, others 44 | Mid-face 542, periphery of the face 383, neck 62, scalp 11, trunk 18, limbs 101, others 14 | Pink/red 858, purple 209, mixed 3, thick 10 | - | 5 mg/kg injected intravenously for 20 min, 532 nm green LED light 10 min after infusion, power density of 80-95 mw/cm^2^, energy density of 96-115 J/cm^2^ | - | 2-3 | - | - | 7.0 ± 6.0 | Improvement |
| Zhu | 2023 | China | 2017.06-2022.01 | Retrospective | 402 | - | ≤ 3 187, 4-6 122, 7-14 93 | - | Head/neck | - | - | 5 mg/kg injected within 5 min, 532 nm green LED light 3 min after injection for 18-25 min, power density ranging 70-90 mw/cm^2^ | 1-3 | 2-3 | - | - | 2-3 | Improvement |
| Liu | 2022 | China | 2017.08-2021.07 | Retrospective | 261 | 114/147 | < 3 73, 3-6 147, 7-13 41 | 0 | Central face 104, peripheral face 156, neck 20, extremity/trunk 41 | Pink 31, red 245, purple 37, hypertrophy 8 | ≤ 25 55, 25-100 103, > 100 163 | 5-5.5 mg/kg intravenously, 532 nm light for 18-25 min, power density of 75-110 mw/cm^2^ | 1: 161, 2: 89, ≥ 3: 71 | - | III, IV | - | - | Improvement |
| Peng | 2022 | China | 2019.01-2020.12 | Retrospective | 11 | - | 2-10 | - | - | - | > 10 | 5 mg/kg intravenously within 5-20 min, 532 nm green LED light 5-10 min after infusion for 20-30 min, power density of 80-100 mw/cm^2^, energy density of 96-120 J/cm^2^ | 2 | 2-6 | - | - | 1-3 | Improvement |
| Tao | 2022 | China | 2019.01-2020.12 | Prospective | 80 | 47/33 | 6.63 ± 1.49 | 0 | Face | - | - | - | - | - | - | - | - | Improvement, scar |
| Zhang_1 | 2022 | China | 2018-2019 | Retrospective | 107 | 48/59 | < 3 35, 3-6 41, 7-14 31 | PDL≥5 sessions | Central of face 63, lateral of face 43, neck 3 | Pink 27, red 57, purple 23 | - | 5 mg/kg intravenously over 20 min, 532 nm LED green light 5-10 min after infusion for 20 min, power density of 80-100 mw/cm^2^, energy density of 90-190 J/cm^2^ | 1: 107, 2: 65 | 2 | - | - | 21.3 (14-33) | Improvement, edema, crust, scar, hyperpigmentation, hypopigmentation |
| Zhang_2 | 2022 | China | 2017.12-2020.05 | Retrospective | 216 | 98/118 | 1-3 84, 3-6 47, 6-9 33, 9-12 29, 12-14 23 | - | Face 180, neck 14, arm/leg 22 | Pink 66, purple 150 | < 5 45, 5-10 134, ≥ 10 37 | 5 mg/kg injection for 10 min, 532 nm LED green light for 20-25 min, power density of 70-80 mw/cm^2^ | 2 | 2 | III, IV | Cold ice pack | ≥ 12 | Improvement, edema, crust, scar, pustules |
| Huang | 2021 | China | 2017.12-2020.10 | Retrospective | 140 | - | 1-5 96, 6-17 44 | - | Face | Red 132, purple 8 | - | 5-7.5 mg/kg intravenously for 20 min, 532 nm LED light 10 min after injection for 18-25 min, power density ranging 80-100 mw/cm^2^ | 1-6 | 2 | III, IV | - | - | Improvement |
| Tan | 2021 | China | 2017.07-2020.01 | Retrospective | 439 | 178/261 | 1-2 268, 3-6 128, 7-14 43 | - | Face 332, neck 45, trunk and extremities 62 | Pink 42, red 351, purple 46 | ≤ 25 71, 25-100 211, > 100 157 | 5 mg/kg injected intravenously, 532-nm green LED light for 20-25 min, power density of 80-85 mw/cm^2^, energy density of 96-115 J/cm^2^ | 1: 147, 2: 83, 3: 78, ≥ 4: 131 | 2 | - | Ice compress for 30 min after irradiation | 2 | Improvement, edema, crust, scar, purpura, hyperpigmentation |
| Khalaf | 2020 | China | 2017.03-2018.06 | Prospective | 6 | - | 6-17 | - | - | - | - | 5 mg/kg injected intravenously for 10 min, 532 nm LED light for 15 min, energy density of 80-110 J/cm^2^ | 3 | - | III, IV | Cold spraying or cold compress for 20 min after treatment | - | Improvement |
| Zhang | 2019 | China | 2017.03-2017.11 | Retrospective | 100 | 60/40 | 1.74 (1-3) | PDL 60, PDT 11, PDL+PDT 5 | Forehead 15, eye socket 43, nose 16,below eye socket 58, mouth 50, chin 18, cheek 73, cheekbone 66, temple 31, vertex 6, ear 4, occiput 10, neck 15 | Pink 35, purplish red 65 | - | 5 mg/kg injected, 532 nm LED green light, power density pf 75-95 mw/cm^2^ | - | - | - | - | 2 | Edema, crust, scar, purpura, hyperpigmentation |
| Zhang | 2014 | China | 2004.05-2010.01 | Retrospective | 132 | 45/87 | 6.92 ± 3.99 | - | Face | Red 89, purple 43 | - | 3.5 mg/kg injected intravenously, copper vapour laser light immediately after infusion for 20-25 min, power density of 80-100 mw/cm^2^, energy density of 120 J/cm^2^ | 1 | - | - | Ice cold bag | 2 | Improvement, scar, hyperpigmentation, hypopigmentation |

Abbreviation: F: Female; FU: Follow up; LED: Light emitting diode; M: Male; PDL: Pulsed dye laser; PDT: Photodynamic therapy; y: Year. Note: The numbers after the age in the age column represent the number of people, and the numbers in the Pre-treatment, Location, and Type columns represent the number of people. Notes: The number immediately following the symbol in the Age column is the age, followed by the number of people; The numbers in the Pre-treatment, Location, and Type columns all represent the number of people; The number immediately following the symbol in the Size column is the range of the PWS, followed by the number of people.

**Table S3 Results of meta-regression analysis**

| **Variable** | **Coef** | **Std. Err** | ***P*** | **95% CI** |
| --- | --- | --- | --- | --- |
| **Improvement ≥ 60%** |  |  |  |  |
| Age, years |  |  |  |  |
| 3-6 vs 0-3 | -0.022 | 0.190 | 0.912 | -0.435, 0.392 |
| 6-18 vs 0-3 | -0.085 | 0.188 | 0.660 | -0.494, 0.325 |
| Sex |  |  |  |  |
| Female vs male | 0.044 | 0.087 | 0.661 | -0.329, 0.417 |
| Session |  |  |  |  |
| 2 vs 1 | 0.184 | 0.258 | 0.514 | -0.531, 0.900 |
| ≥ 3 vs 1 | 0.108 | 0.240 | 0.676 | -0.558, 0.773 |
| Location |  |  |  |  |
| Neck vs face | 0.200 | 0.155 | 0.254 | -0.199, 0.600 |
| Trunk/extremities vs face | -0.455 | 0.138 | 0.022 | -0.810, -0.100 |
| Type |  |  |  |  |
| Red vs pink | 0.015 | 0.121 | 0.906 | -0.295, 0.325 |
| Purple/hypertrophic vs pink | -0.267 | 0.126 | 0.087 | -0.590, 0.056 |
| **Improvement ≥ 75%** |  |  |  |  |
| Age, years |  |  |  |  |
| 3-6 vs 0-3 | -0.047 | 0.066 | 0.493 | -0.203, 0.108 |
| 6-18 vs 0-3 | -0.044 | 0.068 | 0.532 | -0.204, 0.116 |
| Sex |  |  |  |  |
| Female vs male | -0.039 | 0.119 | 0.774 | -0.550, 0.472 |
| Session |  |  |  |  |
| 2 vs 1 | -0.022 | 0.083 | 0.802 | -0.214, 0.170 |
| ≥ 3 vs 1 | 0.170 | 0.104 | 0.141 | -0.070, 0.410 |
| Location |  |  |  |  |
| Neck vs face | -0.025 | 0.232 | 0.920 | -0.764, 0.713 |
| Trunk/extremities vs face | -0.236 | 0.185 | 0.292 | -0.826, 0.354 |
| Type |  |  |  |  |
| Red vs pink | -0.211 | 0.203 | 0.356 | -0.774, 0.351 |
| Purple/hypertrophic vs pink | -0.220 | 0.221 | 0.377 | -0.834, 0.394 |

Abbreviation: Coef: Coefficient; Std. Err: Standard error; CI: Confident interval.
